# Mapping Antimicrobial Resistance in *Escherichia coli* and *Klebsiella pneumoniae* from Complicated Urinary Tract Infections in Oman: Phenotypic and Genotypic Insights

**DOI:** 10.3390/diagnostics15091062

**Published:** 2025-04-22

**Authors:** Nawal AL Shizawi, Zaaima AL Jabri, Fatima Khan, Hiba Sami, Turkiya AL Siyabi, Zakariya AL Muharrmi, Srinivasa Rao Sirasanagandla, Meher Rizvi

**Affiliations:** 1 Department of Microbiology, Suhar Hospital, Ministry of Health, Sohar 100, Oman; nawalalshizawi_22@outlook.com; 2 Department of Microbiology and Immunology, College of Medicine and Health Sciences, Sultan Qaboos University and Sultan Qaboos University Hospital, Muscat 123, Oman; zaeema@squ.edu.om (Z.A.J.); sturkiya@squ.edu.om (T.A.S.); almuharrmi@gmail.com (Z.A.M.); 3 Department of Microbiology, Jawaharlal Nehru Medical College, Aligarh Muslim University, Aligarh 202001, India; fatimasalmanshah@gmail.com (F.K.); hibasamizafar@gmail.com (H.S.); 4 Department of Human and Clinical Anatomy, College of Medicine and Health Sciences, Sultan Qaboos University, Muscat 123, Oman; srinivasa@squ.edu.om

**Keywords:** *E. coli*, *K. pneumoniae*, complicated UTI, whole genome sequencing, AMR, fosfomycin, nitrofurantoin

## Abstract

**Background:** Mapping the local etiology and susceptibility of common pathogens causing complicated urinary tract infection (cUTI) is important for promoting evidence-based antimicrobial prescribing. Evaluating the prevalence of extended-spectrum beta-lactamase (ESBL), AmpC beta-lactamase (AmpC), and carbapenemase-producing *Enterobacterales* (CPEs) is equally important as it informs treatment guidelines and empiric management. Whole genome sequencing (WGS) enhances antimicrobial resistance (AMR) surveillance by complementing phenotypic antimicrobial susceptibility testing, offering deeper insights into resistance mechanisms, transmissions, and evolutions. Integrating it into routine AMR monitoring can significantly improve global efforts to combat antimicrobial resistance. **Methods:** Antimicrobial susceptibility profiles of isolates from cUTI were collected from patients presenting with Sultan Qaboos University Hospital, Muscat and Suhar Hospital, Suhar, Oman. Automated systems as well as manual methods were used for detection of ESBL, AmpC, and CPE. ESBLs, AmpC β-lactamases, and CPEs were further detected by manual methods: double-disk synergy test for ESBL; disk approximation assay and D69C AmpC detection set for AmpC, and mCIM and *KPC/IMP/NDM/VIM/OXA-48* Combo test kit for CPE. WGS was carried out in 11 FOX-resistant *E. coli* and (22 carbapenem-resistant *K. pneumoniae*) isolates with varying susceptibilities to identify circulating clades, AMR genes, and plasmids. Bioinformatic analysis was performed using online tools. **Results:** The susceptibility patterns of *E. coli* from cUTI were as follows: nitrofurantoin (96%), fosfomycin (100%), fluoroquinolones (44%), aminoglycosides (93%), piperacillin-tazobactam (95%), and carbapenems (98%). In comparison, susceptibility rates of *K. pneumoniae* were far lower: nitrofurantoin (38%), fosfomycin (89%), aminoglycosides (82%), piperacillin-tazobactam (72%), and carbapenems (83%). *K. pneumoniae,* however, was more susceptible to fluoroquinolones at 47% in comparison to *E. coli.* The prevalence of ESBL among *E. coli* and *K. pneumoniae* was 37.2% and CRE was 6.2% while the estimated prevalence of AmpC was 5.4%. It was observed that *E. coli* was the predominant ESBL and AmpC producer, while *K. pneumoniae* was the major carbapenem-resistant *Enterobacterales* (CREs) producer. No predominant multi-locus sequence typing (MLST) lineage was observed in AmpC-producing *E. coli* with nine *E. coli* MLST lineages being identified from eleven isolates: *ST-10*, *ST-69*, *ST-77*, *ST-131*, *ST-156*, *ST-167*, *ST-361*, *ST-1125,* and *ST-2520*. On the other hand, a less diverse MLST spectrum (*ST-2096*, *ST-231*, *ST-147*, *ST-1770,* and *ST-111*) was observed in the CRE *K. pneumoniae*. Among the five MLST lineages, *ST-2096* (twelve isolates) and *ST-147* (seven isolates) predominated. WGS revealed that *DHA-1* was the predominant plasmid-mediated *AmpC* gene in *E. coli*, while *OXA-232* and *NDM-5* were the most common carbapenemase genes in *K. pneumoniae*. All *E. coli DHA-1*-positive isolates co-harbored the quinolone resistance gene *qnrB4* and the sulfonamide resistance gene *sul1* while no aminoglycoside resistance genes were detected. The majority of CPE CRE *K. pneumoniae* carried other β-lactamase genes, such as *blaCTX-M-15*, *blaSHV*, and *blaTEM*; all co-harbored the quinolone resistance gene *OqxAB;* and 77% carried the aminoglycoside resistance gene *armA*. **Conclusions:** Our results suggest that fosfomycin is an excellent empiric choice for treating complicated cystitis caused by both *E. coli* and *K. pneumoniae,* while nitrofurantoin is an appropriate choice for *E. coli* cystitis but not for *K. pneumoniae*. Aminoglycosides and piperacillin-tazobactam are excellent intravenous alternatives that spare carbapenems. *DHA-1* was the predominant AmpC in *E. coli,* while *OXA-232* and *NDM-5* were the predominant carbapenemases in *K. pneumoniae*. In AmpC-producing *E. coli,* no MLST predominated, suggesting a significant flux in *E. coli* with lack of stable clades in this region. In contrast, *ST-2096* and *ST-147* predominated in CRE *Klebsiella pneumoniae,* suggesting a stable circulation of these in Oman. WGS profiling provides a deeper understanding of the genetic basis of resistance and enhances surveillance and offers comprehensive insights into pathogen evolution and transmission patterns.

## 1. Introduction

Urinary tract infections (UTIs) rank among the most prevalent bacterial infections worldwide, affecting millions annually and imposing significant healthcare and economic burdens [1,2]. Complicated urinary tract infections (cUTIs) are associated with structural, functional, or metabolic abnormalities of the genitourinary tract and are linked with greater resistance [3]. The increasing prevalence of antimicrobial resistance (AMR) in these pathogens has led to reduced treatment efficacy, longer hospital stays, and higher morbidity. While there are numerous global reports worldwide pertaining to the antimicrobial susceptibility patterns in simple UTIs, there are few that report susceptibility rates in complicated UTIs [3,4]. Relying on local antibiograms of uncomplicated UTIs to treat cUTIs may lead to inappropriate empirical therapy leading to poor outcomes as cUTIs have an increased risk of being associated with multidrug-resistant (MDR) pathogens and extensively drug-resistant (XDR) pathogens [5,6]. Hence, antibiograms specific to cUTIs are essential for optimum empirical therapy [3].

While antimicrobial susceptibility testing provides phenotypic resistance profiles, it does not identify specific genetic determinants, making it difficult to track resistance evolution or predict resistance before it manifests. Phenotypic methods may not detect low-expression resistance genes, silent mutations, or emerging mechanisms of resistance. WGS offers a more comprehensive approach, enabling the identification of AMR genes, horizontal gene transfer events, and clonal relationships between strains. Comprehensive WGS profiling of cUTIs will shed much needed light about AMR evolution at the genomic level in this region.

MDR is defined as acquired non-susceptibility to ≥1 agent in three or more different classes of antimicrobials, while XDR is defined as being non-susceptible to ≥1 agent in all but remaining susceptible to two or fewer antimicrobial classes [7]. These infections are predominantly caused by Gram-negative bacilli, including *E. coli* and *K. pneumoniae*, which frequently exhibit varying levels of resistance through mechanisms such as extended-spectrum beta-lactamase (ESBL) production, AmpC beta-lactamase, and carbapenemase activity [8,9].

The lack of diagnostic precision in identifying these resistance markers often leads to inappropriate antibiotic prescriptions, further fueling resistance. As WGS becomes more accessible, its application is poised to bridge existing knowledge gaps in AMR surveillance, ultimately contributing to improved patient outcomes and public health strategies. Routine identification of the resistance mechanisms will undoubtedly promote antimicrobial stewardship and judicious use of reserve antibiotics.

This study (i) evaluates the susceptibility profile of *E coli* and *K. pneumoniae* isolated from cUTIs to inform practice, (ii) identifies cheap, simple, and reliable tools to detect extended-spectrum beta-lactamases (ESBL), AmpC beta-lactamases and carbapenemase-producing *Enterobacterales* (CPEs) in resource-constrained settings, and (iii) identifies the circulating MLST, AMR genes, and plasmids in AmpC- and carbapenemase-producing strains by whole genome sequencing of representative isolates in Oman.

## 2. Materials and Methods

### 2.1. Study Period and Setting

This study was conducted from September 2022 to August 2023 at the Department of Microbiology and Immunology, College of Medicine and Health Sciences, Sultan Qaboos University, Muscat, Oman, in collaboration with Clinical Microbiology Laboratories at Sultan Qaboos University Hospital (SQUH) and Suhar Hospital, Oman. Ethical approval was obtained from the Medical Research Ethics Committee (MREC), College of Medicine & Health Sciences, Sultan Qaboos University (REF. NO. SQU-EC/377/2021), and the Health Studies and Research Approval Committee, Ministry of Health (MoH/CSR/21/24496).

### 2.2. Study Design

The study evaluated the demographic and antimicrobial susceptibility profiles of *E. coli* and *K. pneumoniae* isolated from cUTI cases in both the hospitals. UTIs in pregnant women, men, children, and patients with an indwelling urinary catheter, renal disease, immunocompromised status, or with an anatomical or functional abnormality of the urinary tract were categorized as cUTIs. ESBL, AmpC, and carbapenemase production were identified both by automated systems as well as manual methods. Genomic profiling was carried out in representative AmpC and CRE isolates to acquire in-depth knowledge of the spectrum of resistance harbored and the prevalent clades and plasmids carried.

### 2.3. Inclusion and Exclusion Criteria

Inclusion criteria: Consecutive, non-duplicate *E. coli* and *K. pneumoniae* isolated from cUTI cases were included in the study.

Exclusion criteria: *E. coli* and *K. pneumoniae* isolated from patients presenting with simple UTIs, infectious etiology other than *E. coli* and *K. pneumoniae* in cUTI.

### 2.4. Sample Size

Over 1194 samples were received from cases of cUTI over the period of one year.

### 2.5. Bacterial Identification and Susceptibility Testing

All the clinical samples were processed as per standard guidelines. Bacterial identification was performed using MALDI-TOF MS (Bruker, Munich, Germany) and Phoenix™ (BD Diagnostics, Franklin Lakes, NJ, USA) automated systems at SQUH, while VITEK 2 (Biomérieux, Tokyo, Japan) was employed at Suhar Hospital as per standard guidelines [10,11,12]. Antimicrobial susceptibility testing was performed and interpreted following CLSI guidelines (2022) [13]. The automated systems identified the isolates as ESBLs. Carbapenem-resistant isolates were subjected to GeneXpert^®^ Carba-R (Cepheid, Sunnyvale, CA, USA) for confirmation of carbapenemase production, while AmpC was inferred on the basis of susceptibility profile and confirmed by manual detection methods.

### 2.6. Manual Phenotypic Detection of ESBL, AmpC, and CPE

We performed manual methods for detection of ESBL, AmpC, and CPE in duplicate.

**Figure 1:**
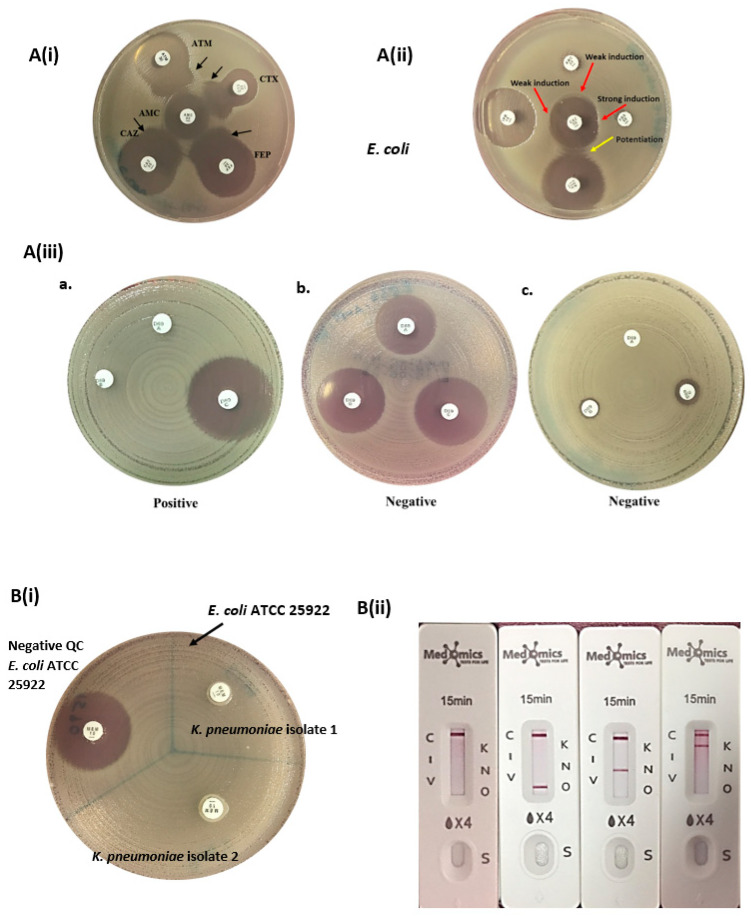
(**A**(**i**)) ESBL **Double-disk synergy test,** (**A**(**ii**)) **disk approximation test, and** (**A**(**iii**)) **D69C AmpC detection set for AmpC detection;** (**B(i**)) **modified carbapenem inactivation method (mCIM) and** (**B**(**ii**)) **Combo test kit for carbapenemase detection;** (**A**(**i**)). Synergistic activity between the substrates and amoxicillin-clavulanic acid is indicated by black arrows. (**A**(**ii**)) Strong and weak induction (red arrows) towards the ceftazidime (CAZ) as substrate and imipenem (IPM), cefoxitin (FOX), amoxicillin-clavulanic acid (AMC), and piperacillin-tazobactam (TZP) as inducers. The potentiation between drugs is indicated by yellow arrows. (**A**(**iii**)) **D69C AmpC detection set.** (**a**) AmpC positive. (**b**,**c**) AmpC negative (exhibiting other type(s) of resistance). Disk A (cefpodoxime 10 μg + AmpC inducer), disk B (cefpodoxime 10 μg + AmpC inducer + ESBL inhibitor), and disk C (cefpodoxime 10 μg + AmpC inducer + ESBL inhibitor + AmpC inhibitors). **Modified carbapenem inactivation method (mCIM)** (**B**(**i**)), identification of carbapenemase-producing strains via mCIM test. The control strain *E. coli* ATCC 25922 was carbapenemase negative, having a zone size of >19 mm. Two target isolates carried carbapenemase, which broke down the 10 μg meropenem after incubation for 4 h ± 15 min at 35 °C. **Combo test kit for carbapenemase detection** (**B**(**ii**))**,** from left to right—carbapenemase not detected (C = control), OXA-48 detected (O = OAX-48), NDM detected (N = NDM), and KPC detected (K = KPC) (V = VIM) (I = IMP).

**ESBL Detection**: The double-disk synergy method was utilized for identifying ESBLs. Amoxicillin-clavulanic acid (AMC, 20/10 μg) (Oxoid, Basingstoke, UK) was placed at the center of Mueller–Hinton agar (MHA), and ceftazidime (CAZ, 30 μg) (Oxoid, UK), cefepime (FEP, 30 μg) (Oxoid, UK), aztreonam (ATM, 30 μg) (Oxoid, UK), cefotaxime (CTX, 30 μg) (Oxoid, UK), cefuroxime (CXM, 30 μg) (Oxoid, UK), cefoxitin (FOX, 30 µg) ceftriaxone (CRO, 30 μg) (Oxoid, UK), cefoperazone (CFP, 75 μg) (Liofilchem, Roseto degli Abruzzi, Italy), and cefpodoxime (CPD, 30 μg) (Liofilchem, Italy) were placed at a distance of 20 mm (edge to edge) from AMC. The synergistic activities between AMC and β-lactam antibiotics were observed after incubating the plates at 35 ± 2 °C for 18–24 h. Isolates were confirmed as ESBL producers when a clear extension of the edge of the inhibition zone of any of the β-lactam antibiotics was observed towards AMC or when a zone of inhibition appeared in between β-lactam antibiotic and AMC, Figure 1A(i) [14,15].

**AmpC detection:** Two methods were utilized for detection of AmpC beta-lactamases: disk approximation test and AmpC detection set (D69C).

**Disk approximation test:** This was an in-house test. A ceftazidime disk (CAZ, 30 μg) (Oxoid, UK) was placed in the center of the inoculated MHA and disks of the inducing substrates: imipenem (IPM, 10 µg), amoxicillin-clavulanic acid (AMC 20/10 µg), and piperacillin-tazobactam (TZP 110 µg) were placed at a distance of 20 mm apart (edge to edge) from a ceftazidime disk, while the distance between cefoxitin (FOX, 30 µg) and ceftazidime disk was adjusted to 15 mm apart (edge to edge) to attain better induction [16,17]. The plates were incubated at 35 °C ± 2 for 18–24 h. AmpC was identified by the flattening of the zone of inhibition towards ceftazidime, as seen in Figure 1A(ii).

**AmpC detection set (D69C):** This is a commercial AmpC detection set from Mast Group Ltd., Bootle, UK. The set contained three cartridges A, B, and C. The disk content of cartridge A is cefpodoxime (10 µg) with AmpC inducer; in cartridge B, the disk contains cefpodoxime (10 µg) with AmpC inducer and ESBL inhibitor; and in cartridge D, the disk composed of cefpodoxime (10 µg) with AmpC inducer, ESBL inhibitor, and AmpC inhibitors. The inducers and inhibitors in each cartridge were unspecified. The procedure was performed and interpreted as per the manufacturer’s instructions [18] as seen in Figure 1A(iii).

**Carbapenemase detection:** Two methods were utilized for detection of CPE: modified carbapenem inactivation method and a lateral flow immunochromatographic assay.

**Modified carbapenem inactivation method (mCIM):** The modified carbapenem inactivation method (mCIM) was performed as per CLSI 2023 [19] as seen in Figure 1B(i).

**Lateral flow immunochromatographic assay:** KPC/IMP/NDM/VIM/OXA-48 Combo test kit (Medomics, Nanjing, China) is a lateral flow immunochromatographic assay (LFIA) developed for the specific detection of the five most common carbapenemases (*KPC*, *IMP*, *NDM*, *VIM,* and *OXA-48*) using a double-antibody sandwich assay. The procedure was performed, and the results were interpreted according to the manufacturer’s instructions. Figure 1B(ii) shows some of the results obtained.

### 2.7. Whole Genome Sequencing (WGS)

As WGS enhances AMR surveillance by complementing phenotypic AST, offering deeper insights into resistance mechanisms, transmissions, and evolutions, we carried out this baseline study in cUTI in Oman.

The isolates for whole genome sequencing were collected from Sultan Qaboos University and Sohar Hospital. Both cater to a diverse population representative of many regions of Oman. Since we have limited information on AmpC and CRE in cUTI, we selected isolates with varying susceptibility profiles in order to obtain a heterogenous flavor of the circulating clades.

Thus, representative 11 AmpC and 22 CRE *E. coli* and *K. pneumoniae* strains were subjected to WGS. DNA was extracted from an overnight culture using the QIAamp DNA Mini Kit (Qiagen, Hilden, Germany) for whole genome sequencing. The manufacturer’s protocol was followed with slight modifications. For highly mucoid *K. pneumoniae* isolates, a pre-lysis step was performed, which included the preparation of a pre-lysis buffer consisting of 100 μL of Tris//EDTA (TE) buffer (ThermoFisher Scientific, Waltham, MA, USA), 1.00 μL lysozyme 10 mg/mL (final concentration 0.1 mg/mL) (Sigma-Aldrich, St. Louis, MO, USA), 0.20 μL lysostaphin 10 mg/mL (final concentration 0.02 mg/mL) (ThermoFisher Scientific, USA), and 0.10 μL RNAse A 100 mg/mL (final concentration 0.1 mg/mL) (Qiagen, Hilden, Germany). The bacterial suspension from an overnight culture was centrifuged at 8000× *g* for 2 min, and the pelleted bacterial cells were resuspended in 100 μL of the pre-lysis buffer by pipetting up and down several times with a P200 pipette. The suspension was then incubated for 60 min. After incubation, 1.00 μL of Proteinase K (Sigma-Aldrich, St. Louis, MO, USA) was added as a final step in the pre-lysis stage. The sample was then ready for the first step of the Qiagen DNA extraction protocol. After DNA quantification by NanoDrop (ThermoFisher Scientific 1000 NanoDrop Spectrophotometer), the samples were sent to microbes NG in the UK for WGS by Illumina next-generation sequencing (https://microbesng.co.uk, Birmingham, UK) accessed on 3 October 2023. The DNA samples were prepared and then sequenced following the manufacturer’s protocol. ILLUMINA SEQUENCING (SGS and EGS) Genomic DNA libraries are prepared using the Nextera XT Library Prep Kit (Illumina, San Diego, CA, USA) following the manufacturer’s protocol with the following modifications: input DNA is increased two-fold, and PCR elongation time is increased to 45 s. DNA quantification and library preparation are carried out on a Hamilton Microlab STAR automated liquid handling system (Hamilton Bonaduz AG, Bonaduz, Switzerland). Pooled libraries are quantified using the Kapa Biosystems Library Quantification Kit for Illumina. Libraries are sequenced using Illumina sequencers (HiSeq/NovaSeq, Illumina, San Diego, CA, USA) using a 250 bp paired-end protocol. Reads are adapter trimmed using Trimmomatic 0.30 with a sliding window quality cutoff of Q15. De novo assembly is performed on samples using SPAdes version 3.7 [20], and contigs are annotated using Prokka 1.11 [21]. The assembled and annotated sequences were uploaded to the website to carry out further analysis.

Detailed resistance gene profiling, with bioinformatics analysis, was conducted using Center for Genomic Epidemiology (CGE) website (https://www.genomicepidemiology.org/) accessed on 17 November 2023 for identifying multi-locus sequence typing (MLST) using MLST tool [22], plasmids using PlasmidFinder tool [23], and antibiotic resistance genes using ResFinder tool [24]. All the genome sequences were submitted to NCBI and accession numbers were awaited. The bio sample accessions are provided in Supplementary Document S1. *E. coli* MG 1655 RJF293 (GenBank accession NZ_JARVXJ000000000, https://www.ncbi.nlm.nih.gov/nuccore/) [accessed on 4 December 2023] and *K. pneumoniae* RJF293 (GenBank accession number CP014008, https://www.ncbi.nlm.nih.gov/nuccore/) [accessed on 4 December 2023] references were used. iTOL was used to visualize and annotate the tree.

### 2.8. Statistical Analyses

Data were analyzed using SPSS (version 23). Continuous data were expressed as mean ± SD, while categorical variables were presented as percentages. Sensitivity and specificity analyses of diagnostic tests were performed, and results were visualized using Microsoft Excel.

## 3. Results

### 3.1. Demographic Characteristics

Among the 1194 non-duplicate cases of cUTIs, the majority, 60% (712), were females and 40% (482) males. cUTI predominance was observed in the >60 year age group (47.2%), followed by 30–60 year (32.7%) and ≤13 year (10.7%) age groups. Key complicating factors were urinary instrumentation, hypertension, diabetes, chronic kidney disease, dyslipidemia, sickle cell anemia, malignancy, renal stones, urinary obstruction, benign prostatic hyperplasia, and thalassemia. A total of 1233 bacterial isolates were identified, among which *E. coli* predominated, 406 (33%) followed by *K. pneumoniae* 163 (13.5%). *E. coli* predominated among both adults (35.5%) and children (49.4%). In catheter specimen urine samples, *Candida* spp. predominated in adults (41.9%).

### 3.2. Antimicrobial Susceptibility Profiles

*E. coli* exhibited excellent susceptibility to fosfomycin (100%), nitrofurantoin (96%), piperacillin-tazobactam (95%), gentamicin (87%), amikacin (98%), and meropenem (99%). *K. pneumoniae* had lower susceptibility rates than *E. coli*, with significantly low (38%) susceptibility to nitrofurantoin. Fosfomycin rates were 89%, meropenem and amikacin 84%, and gentamicin 80%, as shown in Table 1.

The susceptibility rates to cephalosporins was slightly lower in *E. coli* [cefazolin (27%), cefuroxime (51%), ceftriaxone (54%), ceftazidime (57%), and cefepime (56%)] than in *K. pneumoniae* [cefazolin (33%), cefuroxime (55%), ceftriaxone (61%), ceftazidime (60%), and cefepime (63%)]. Similar susceptibilities to amoxicillin-clavulanic acid (66% and 65%) and cotrimoxazole (63% and 65%) were observed in both *E. coli* and *K. pneumoniae,* respectively. Lower susceptibility was observed for ciprofloxacin (44%) and levofloxacin (46%).

The manual phenotypic methods for detection of ESBL and CPE fully concurred with the results obtained by automated systems. The D69C method better detected de-repressed AmpC, while the disk approximation test was equally good in detecting inducible AmpC.

### 3.3. Prevalence of ESBL, AmpC Beta-Lactamases, and Carbapenemases in cUTI

**ESBL:** The overall prevalence of ESBL among *E. coli* and *K. pneumoniae* (*n* = 569) was 37.2%, with *E. coli* accounting for 40.4% and *K. pneumoniae* 20.2% (Table 2). The ESBL prevalence increased to 43.8% in *E. coli* and 20.8% when isolates co-harboring ESBL and AmpC were included.

**AmpC**: AmpC alone was identified in 16 (2.8%) isolates, while AmpC and ESBL co-carriage was identified in 15 (2.6%) cases.

**Carbapenemases**: The overall prevalence of carbapenemase producers was 35 (6.2%). *K. pneumoniae* was the predominant carbapenemase producer, accounting for 17.2% of the total *K. pneumoniae* isolated, compared to *E. coli* 1.7%, as shown in Table 2.

### 3.4. In Silico Identification of Resistance Genes from Genomic Sequences of AmpC-Producing E. coli

The MLST analysis of the AmpC strains identified the presence of nine *E. coli* lineages: *ST-10*, *ST-69*, *ST-77*, *ST-131*, *ST-156*, *ST-167*, *ST-361*, *ST-1125,* and *ST-2520*, with *ST-10* and *ST-69* being observed in two isolates each.

Diverse antimicrobial resistance genes were detected in the AmpC-producing *E. coli*. Four beta-lactam resistance genes *blaCTX-M-15*, *blaOXA-1*, *blaTEM-1B,* and *blaTEM-35* were identified (Table 3). The *blaDHA-1* gene encoding the *DHA-1* AmpC beta-lactamase was the sole AmpC gene identified in the nine isolates. Among the two FOX-resistant isolates that tested negative for *AmpC* genes, one co-harbored *blaCTX-M-15* and *blaOXA-1*, and the other carried *blaCTX-M-15*, *blaTEM-1B,* and *blaTEM-35* (Table 3). Three AmpC producers were resistant to piperacillin-tazobactam, one carried *blaOXA-1* while the others co-harbored several β-lactamases. The predominant quinolone resistance gene was *qnrB4*, while the other two were *aac(6′)-Ib-cr* and *qnrS1*. Most isolates carried one of the quinolone resistance genes. Five ciprofloxacin susceptible isolates also carried the *qnrB4* gene. The tetracycline resistance genes *(tet(A*) and *tet(B)*) were detected in seven isolates with *tet(A)* being the most frequent. Aminoglycoside resistance was infrequent with one isolate carrying *aac(3)-IIa* and *aadA5* and the other *aph(3″)-Ib* and *aph(6)-Id*.

The *marA* gene, which is responsible for antibiotic efflux and reduced cellular permeability to antibiotics, was carried by all but one isolate. The most frequently detected efflux pump genes belonged to resistance–nodulation–cell division (RND) and major facilitator superfamily (MFS) efflux gene families (Supplementary Table S1). H-NS, which confers resistance to macrolides, fluoroquinolones, cephalosporins, cephamycins, penams, and tetracyclines, predominated among the efflux genes followed closely by *evgA*, *TolC,* and *emrB*. Four antibiotic target alteration genes were detected (*bacA*, PmrF, *ugd,* and *eptA*), among which *PmrF* predominated.

**Plasmid identification in *E. coli:*** Ten different plasmids were detected among the eleven FOX-resistant *E. coli* with nine having one or more plasmids and two with none. *IncFIB(AP001918)* predominated (7/11) followed by *IncFII (5/11)*, *IncFIA (3/11), Col(BS512)*, *IncFII(pRSB107),* and *IncI1-I(Alpha)* identified in two isolates each. The other four plasmids, *Col(IMGS31)*, *IncB/O/K/Z*, *IncY,* and *p0111,* were detected in isolated strains.

### 3.5. In Silico Identification of Resistance Genes from Genomic Sequences of Carbapenemase-Producing K. pneumoniae

Among the five MLST lineages identified, (*ST-2096*, *ST-231*, *ST-147*, *ST-1770,* and *ST-11)*, *ST-2096* (12/22) and *ST-147* (7/22) predominated. The ST-1770 was identified as *Klebsiella quasipneumoniae*. The resistance genes’ heat map was prepared utilizing ResFinder, as shown in Table 4.

The β-lactam resistance genes predominated with as many as 18 different β-lactam resistance genes being identified (blaSHV-11, blaSHV-28, blaSHV-40, blaSHV-56, blaSHV-67, blaSHV-89, blaSHV-106, blaTEM-1, blaCTX-M-8, blaCTX-M-15, blaDHA-1, blaOXA-1, blaOXA-9, blaOXA-48, blaOXA-232, blaNDM-5, blaKPC-2, and blaOKP-B-3).

Among the carbapenemases, *blaOXA-232* predominated (11/22), followed by *blaNDM-5* (5/22). Single isolate carriage of *blaOXA-48* and *blaKPC-2 (ST-1770*) was observed. All *ST-2096 K. pneumoniae* carried *blaCTX-M-15*, *blaOXA-1*, *blaSHV-28,* and *blaSHV-106* while *ST-147* carried *blaSHV-11* and *blaSHV-67*. Co-carriage of *bla_DHA-1_* and *bla_CTX-M-8_*, *bla_SHV-40_*, *bla-_SHV56_*, and *bla-_SHV89_* was observed in a carbapenem-resistant CPE-negative isolate. One carbapenem-resistant isolate (*K. pneumoniae* 1776) was notable for not carrying any carbapenemase genes. On the contrary, it carried *blaDHA-1*, *blaCTX-M-8*, *blaSHV-40*, *bla-SHV56,* and *bla-SHV89*. Eight aminoglycoside resistance genes (*aac(6′)-Ib*, *aadA1*, *aadA2*, *aph(3′)-Ia*, *ph(3′)-VI*, *aph(6)-Id*, *armA,* and *rmtF*) were identified, and the majority (17/22) carried the *armA* gene. The *rmtF* gene was detected only in one isolate. Eighteen isolates resistant to gentamicin, netilmicin, and amikacin harbored 16S rRNA methyltransferase (17 carried *armA*, 1 carried *rmtF*).

Six fluoroquinolone resistance genes were identified: *aac(6′)-Ib-cr*, *qnrA*, *qnrB*, *qnrS*, *OqxA,* and *OqxB*. All isolates carried *OqxA* and *OqxB,* and 13 co-harboured *aac(6′)-Ib-cr*. The *mdtq* gene, responsible for antibiotic efflux and reduced cellular permeability, was detected in all isolates except one (Supplementary Table S2). Four additional efflux pumps, *lptD*, *Klebsiella pneumoniae kpnE*, *Klebsiella pneumoniae kpnF* (22/22), and *qacEdelta1* (18/22), were detected by RGI.

**Plasmid profiling in *K. pneumoniae*:** In the CPE *K. pneumoniae*, seventeen different plasmids were identified with each strain carrying one or more plasmids with one isolate remarkably carrying seven different plasmids. Overall, *IncFIB (pNDM-Mar*) and *IncHI1B (pNDM-MAR)* predominated, found in 16/22 isolates each, followed by *IncFIB (K*) (15/22), *ColKP3* (11/22), *IncFIB (pKPHS1*) (6/22), *IncR* (5 isolates), *IncFIB (pQil)* (3/22), and *repB(R1701)* (2/22). The remaining plasmids, *IncFIB(K)*, *Pcav 1099-114*, *InFII*, *InFII(K)*, *InFII(pCRY)*, *IncI1-I(Alpha)*, *IncN*, *IncX3*, *IncX4,* and *Col4401* were detected in isolated strains.

## 4. Discussion

Mapping antimicrobial susceptibility of uropathogens in cUTI is imperative in order to institute optimum empiric treatment. cUTIs are at a higher risk of treatment failure, as these infections are frequently associated with MDR Gram-negative bacteria [25]. *E. coli* and *K. pneumoniae*, the two most common uropathogens in cUTIs, are frequently associated with β-lactamases such as ESBLs, AmpC β-lactamases, and carbapenemases [26]. Optimizing the detection of these resistance markers through phenotypic and genotypic tools is critical for appropriate management of cUTIs and for promoting antimicrobial stewardship. Automated systems like Phoenix or Vitek do not detect AmpC beta-lactamases, and manual methods for detecting AmpC may be utilized. A previous study by this group highlighted that these manual methods have good specificity and sensitivity [16].

This study reveals key differences in the susceptibility rates of these two pathogens. *E. coli* exhibited significantly higher susceptibility rates to nitrofurantoin (96%) compared to *K. pneumoniae* (38%). However, both displayed comparable rates to fosfomycin (100% vs. 89%). Babiker et al. (2019) [27] advocated fosfomycin as a good option for management of UTIs. Both nitrofurantoin and fosfomycin cause low collateral damage and have a broad antimicrobial spectrum. Both pathogens exhibited unsatisfactory susceptibility to cotrimoxazole and fluoroquinolones (60% and 50%, respectively), which significantly limits their utility in empiric management. Similar rates were reported by Li et al. (2017) [25]. Susceptibility to cephalosporins was slightly lower in *E. coli* [cefazolin (27%), cefuroxime (51%), ceftriaxone (54%), ceftazidime (57%), and cefepime (56%)] than in *K. pneumoniae* [cefazolin (33%), cefuroxime (55%), ceftriaxone (61%), ceftazidime (60%), and cefepime (63%)], which indicates higher prevalence of ESBL in *E. coli* than in *K. pneumoniae,* which was corroborated by further testing. Mohamed et al. (2020) [28] reported a similar trend.

Conversely, the lower piperacillin-tazobactam, aminoglycoside, and carbapenem susceptibility rates in *K. pneumoniae* [piperacillin-tazobactam 72%, amikacin (84%), gentamicin (80%), ertapenem (81%), imipenem (83%), and meropenem (84%)] compared to *E. coli* [piperacillin-tazobactam (95%), amikacin (98%) gentamicin (87%), ertapenem (98%), imipenem (98%), and meropenem (99%)] suggests that *K. pneumoniae* more frequently carries *blaOXA-1*, aminoglycoside and carbapenem resistance genes. Fernández-Martínez et al., 2018 [29]; Abo-State et al., 2018 [30]; and Arumugam et al., 2024 [31] corroborated these findings.

We observed a clear dominance of ESBL and AmpC in *E. coli* and CPE in *K. pneumoniae*. *E. coli* ESBL prevalence (43.8%) was more than double that of ESBL (20.8%) in *K. pneumoniae*. A study from Egypt reported similar findings: a higher prevalence of ESBL (62–69% in *E. coli*, which was double that in *K. pneumoniae* (30–37%) [32]. AmpC prevalence too was higher in *E. coli* (5.9%) than in *K. pneumoniae* (4.3%). CPE however clearly predominated among *K. pneumoniae* (17.2% vs. 1.7% in *E. coli*). AL Mamari et al., 2022 [3], reported similar prevalence in this region. As in other parts of the world, XDR *K. pneumoniae* is a grave public health concern in Oman [33,34].

A surprisingly pronounced genetic heterogeneity was observed in the AmpC-carrying strains, with nine MLST types observed (*ST-10*, *ST-69*, *ST-77*, *ST-131*, *ST-156*, *ST-167*, *ST-361*, *ST-1125,* and *ST-2520*), indicating a great flux in the circulating *E. coli* clades in this region. Only *ST-167*, *ST-361*, *ST-131*, and *ST-156* have been reported previously from this region [35]. Among these, *ST 131* is a globally ubiquitous ST uropathogenic clade associated with *CTX-M* type of ESBL, while *ST 69* is usually sensitive to most antimicrobials [36,37]. It was interesting to note that all *E. coli* carried plasmid-mediated AmpC gene, *blaDHA-1,* indicating the endemicity of this gene in Oman. Unpublished data from this group support this finding. Similar high prevalence of *blaDHA-1* in uropathogenic *E. coli* have been reported from Egypt, Iran, and India while Saudi Arabia reported predominance of *CMY-2* [38,39,40,41]. It is important to note that there is significant population mobility between Oman, Egypt, Iran, and India, which is mirrored by *blaDHA-1* prevalence in these countries.

The rare carriage of aminoglycoside-modifying/resistance genes support the empirical use of gentamicin and amikacin in managing *E. coli* AmpC-mediated cUTIs, thus sparing carbapenems. On the other hand, quinolones should be prescribed with caution as all isolates carrying *bla DHA-1* harbored the *qnrB4* gene. Similar findings were reported by Kamruzzaman et al. (2013) [42], who reported that *E. coli* isolates carried both *bla_DHA-1_* and *qnrB* genes. Mata et al. (2011) [43] too found a close association between *bla_DHA-1_* and *qnrB* genes. The *aac(6′)-Ib-cr,* a multifunctional gene that induces resistance to both ciprofloxacin and aminoglycosides, was identified in one isolate only [44].

*IncFIB(AP001918)* was the predominant plasmid in *blaDHA-1*-carrying *E. coli*, followed by *IncFII*, *IncFIA*, *Col(BS512)*, *IncFII(pRSB107),* and *IncI1-I(Alpha).* Ingti et al. (2017) [45] reported that *E. coli* harboring the *blaDHA-1 AmpC* gene was associated with several plasmids *IncFIB*, *IncFIA*, *IncK*, *IncL/M*, *IncHI1*, *IncB/O,* and *IncI1*, three of which were found in our study too. Among these, the *IncI1* plasmids are most commonly associated with antimicrobial resistance and are so common that a typing scheme has been proposed by using plasmid multi-locus sequence typing [23]. Plasmids carrying *blaDHA-1* in our study co-harbored *qnrB4*, *mph(A)*, and *sul1* genes, a finding which was corroborated by Hennequin et al., 2018 [46]. Four plasmids, *Col(IMGS31)*, *IncB/O/K/Z*, *IncY,* and *p0111* were identified in one isolate each. Among these, the *p0111* plasmid has been reported to harbor *aadA2*, *drfA12,* and *tetA,* conferring resistance to aminoglycosides, trimethoprim, and tetracycline, respectively [47]. The colicinogenic (*Col*) plasmid type identified in some isolates usually carry colicin, a toxic protein produced by some strains of *E. coli* [48]. In two isolates no plasmids were detected although they carried several AMR genes along with the *DHA-1* gene. They may be encoded by other MGEs such as integrative conjugative elements or by plasmids integrated into the chromosomes of these isolates.

Multi-locus sequence typing identified five ST lineages in the CPE *K. pneumoniae*: *ST-2096*, *ST-231*, *ST-147*, *ST-1770,* and *ST-111,* among which *ST-2096* predominated. These findings are quite different from a previous study in Oman where the majority of XDR/PDR *K. pneumoniae* were *ST-231* [49]. Al Fadhli et al., 2023 [50] reported predominance of *ST-14* and *ST-231* in the Arabian Peninsula with *ST-231* common in Oman and *ST-2096* in Saudi Arabia. The constant flux in this region merits regular surveillance. A study from India reported that *ST-231* was the predominant ST (34.8%), followed by *ST-147* (23.5%) and *ST-14* [51]. Long-term studies delineating evolution and transmission of uropathogenic *K. pneumoniae* are needed to appreciate the changing epidemiology of the circulating clades leading to evolving resistance in this region.

Among the carbapenemases, *bla_OXA-232_* predominated, followed by *bla_NDM-5_*, *bla_OXA-48_*_,_ and *bla_KPC-2_*. All ST-2096 and ST-147 isolates carried the ESBL gene *bla_CTX-M-15_*, which reflects the strong association and co-localization of *bla_OXA232_* and *bla_NDM-5_* with *bla_CTX-M-15_* in these clades, an association also observed in other studies [52,53,54]. ST-231 co-harbored ESBL genes *bla_CTX-M-15_* and *bla-_OXA232_*, which was also reported by Al-Quraini et al. (2022) [49] and Mancini et al. (2018) [55]. All isolates of *ST-2096* carried *bla_OXA-1_* while the majority (6/7) of isolates of *ST-147* harbored *bla_OXA-9_*. Shankar et al. (2022) [53] found that *bla_OXA-1_* integrated in the chromosome of *ST-2096,* and Di Pilato et al. (2022) [56] reported *bla_OXA-1_* and *bla_OXA-9_* in *ST-147*. *K. pneumoniae ST-2096* predominantly carried *OXA-232* carbapenemase, a frequent variant of *OXA-48-*like carbapenemase in Oman, while *ST-147* predominantly carried *NDM-5*. Studies from Qatar and India corroborate our findings [52,57]. It is important to note that current screening for carbapenemases in the diagnostic laboratories in Oman is carried out using the Cepheid GeneXpert CARBA-R test, which includes only five genes (*OXA-48*, *KPC*, *NDM-1*, *VIM*, and *IMI*) and does not detect *OXA-232* or *NDM-5*, potentially leading to underestimation of these variants in routine diagnostics. Among the CPEs, *ST111* alone co-harbored *bla-DHA-1* with *blaCTX-M-8* and importantly did not carry CPE. No co-carriage of plasmid-mediated AmpC beta-lactamases was observed among CPE *K. pneumoniae*.

One isolate of *Klebsiella quasipneumoniae* (*ST-1770*) carrying *KPC-2* was observed in this study. It is considered an emerging pathogen in healthcare settings, which has the propensity to acquire carbapenemase plasmids [58,59,60]. Venkitapathi et al., 2022 [60], have reported this pathogen in recurrent UTIs in women.

Aminoglycoside resistance was mediated by *armA*, *aac(6′)-Ib*, *aadA1*, *aadA2*, *aph(3′)-Ia*, *ph(3′)-VI*, *aph(6)-Id*, and *rmtF.* Alarmingly, the majority (17/22) of the isolates carried the *armA* gene. Among 16S rRNA methyltransferase genes, *armA* and *rmtB* are the most prevalent in Asia [61,62,63]. In contrast, Al-Quraini et al. (2022) [49] detected *armA* and *rmtB* genes only in one isolate, which co-harbored *blaOXA-232* and *bla-NDM-5*. These findings indicate the increasing prevalence of plasmids carrying the *armA* gene among the CPE *K. pneumoniae* in Oman, which may be attributed to the excessive antimicrobial use during the COVID-19 pandemic. This study indicates the emergence of CPE *K. pneumoniae* strains harboring *armA* and *rmtF* genes that lend resistance to all currently available aminoglycosides, which is a cause of great concern.

Fluoroquinolone resistance was mediated by *OqxA* and *OqxB* genes while *ST-2096* co-harbored *aac(6′)-Ib-cr,* and *ST-147* predominantly co-harbored *qnrS*. Shankar et al., 2022 [53] reported similar findings. The *aac(6′)-Ib-cr* gene was found integrated in the chromosome of *K. pneumoniae* of *ST-2096,* which was transferred through MGEs [53]. A study from India reported *qnrB*, *aac(6′)-Ib-cr*, *oqxA*, and *oqxB* in XDR *K. pneumoniae* belonging to *ST147* [64].

The *fosA6* gene was the predominant gene in *ST-2096* and *ST-231* clones, which agrees with previous studies from California and India [65,66]. On the other hand, the *fosA5 gene* was only found in the *ST-147* clone, similar to Al-Quraini et al.’s report. (2022) [49]. Although all isolates harbored the *fosA* gene, only four isolates phenotypically expressed resistance to fosfomycin.

The increasing transmission of CPE through conjugative plasmids is a cause of grave public health concern. Among the seventeen different plasmids identified in the CPE *K. pneumoniae*, *IncFIB (pNDM-Mar)* and *IncHI1B(pNDM-MAR*) predominated followed by *IncFIB(K)*, *ColKP3*, *IncFIB(pKPHS1)*, *IncR*, *IncFIB(pQil),* and *repB(R1701)*. The other nine plasmids, *IncFIB(K)(Pcav1099-114)*, *InFII*, *InFII(K)*, *InFII(pCRY)*, *IncI1-I(Alpha)*, *IncN*, *IncX3*, *IncX4,* and *Col4401,* featured in individual isolates. This contrasted with findings of Piccirilli et al. (2021) [67], who reported predominance of *IncFII(K)* followed by *IncFIB(K)*, *Col (MG828),* and *IncFIA(HI1)*. This highlights the importance of local surveillance of genomic plasmids as studies show there are regional variations in circulation of plasmids. A recent study conducted by Zeng et al. (2022) [68] found that *IncFIB(pNDM-Mar*) and *IncHI1B(pNDM-MAR*) commonly harbored *blaNDM.* Paskova et al. (2018) too reported their role in the dissemination of *blaNDM-1*, *blaSHV-12*, *blaCTXM-15*, and *blaOXA-1* among *K. pneumoniae* in Europe [69]. The IncF plasmids predominated in CPE *K. pneumoniae* in Italy. This plasmid family is widely prevalent in *Enterobacterales* of clinical importance [67].

*ColKP3* replicon exclusively harbored the *bla-OXA-232*, a finding corroborated by Shen et al., 2023 [70], who reported *ColKP3* in all (100%) and *IncFIB-*like in 85.2% CPE *K. pneumoniae ST231* isolates carrying *blaOXA-232* isolates. Interestingly, *IncFIB(pKPHS1)* was only identified in CPE *K. pneumoniae ST147,* which is in agreement with Spadar et al. (2022) [71].

Identifying the circulating MLST, AmpC, β-lactamases, CPE, and plasmids at the national level is essential for tracking the molecular epidemiology and transmission of resistance genes, which will aid in developing effective strategies to control the spread. Developing local-focused antibiograms aids in optimizing empiric antimicrobial therapy and in promoting antimicrobial stewardship.

## 5. Conclusions

Mapping antimicrobial susceptibility of uropathogens causing complicated urinary tract infections (cUTIs) is critical in order to guide empiric antibiotic therapy, particularly due to the growing resistance. This study highlights significant differences in antimicrobial susceptibility patterns between *E. coli* and *K. pneumoniae*. With its broad spectrum of activity, fosfomycin emerged as an excellent choice for the management of cUTI against both pathogens. On the other hand, while nitrofurantoin continues to be an excellent option for *E. coli* cystitis, it demonstrated poor activity against *K. pneumoniae*. Empiric fluoroquinolone therapy is best avoided until susceptibility is confirmed. Regular monitoring of the circulating AMR genes is essential for tracking and controlling the spread of drug-resistant variants. A clear pattern of ESBL and AmpC dominance was observed in *E. coli* and CPE predominance in *K. pneumoniae*. The sole AmpC beta-lactamase gene circulating in this region was *blaDHA-1,* and *OXA-232* and *NDM-5* were the predominant circulating carbapenemases. Significant MLST heterogeneity was observed in AmpC-producing *E. coli* while a distinct predominance of *ST-2096* and *ST-147* was observed in CPE *K. pneumoniae,* pointing to a stable CPE population. The emergence of CPE *K. pneumoniae* strains harboring *armA* and *rmtF* genes, which are resistant to all currently available aminoglycosides, is an alarming development. The dominance of IncF plasmids, such as *IncFIB(AP001918)* and *IncFIB(pNDM-Mar),* underscores their significant role in mediating multidrug resistance, particularly the dissemination of AmpC- and carbapenemase-producing genes among clinical Enterobacterales isolates. Continuous local surveillance of plasmid-mediated antimicrobial resistance is essential along with effective infection prevention control and antimicrobial stewardship measures.

## Figures and Tables

**Table 1:** Susceptibility profile of *E. coli* and *K. pneumoniae* from cUTI cases.

Antibiotics	*E. coli*		*K. pneumoniae*	
	Number of Isolates Tested	Susceptibility%	Number of Isolates Tested	Susceptibility%
Nitrofurantoin	404	96%	157	38%
Cotrimoxazole	408	63%	162	65%
Fosfomycin	330	100%	134	89%
Ampicillin	408	29%	-	-
Cefazolin	256	27%	92	33%
Cefuroxime	402	51%	161	55%
Ceftriaxone	407	54%	161	61%
Ceftazidime	406	57%	162	60%
Cefepime	407	56%	163	63%
Amoxicillin-clavulanate	152	66%	77	65%
Ampicillin-sulbactam	249	65%	103	52%
Piperacillin-tazobactam	408	95%	163	72%
Gentamicin	408	87%	163	80%
Amikacin	407	98%	163	80%
Ciprofloxacin	347	44%	133	47%
Levofloxacin	357	46%	136	48%
Ertapenem	371	98%	146	81%
Imipenem	408	98%	163	83%
Meropenem	406	99%	163	84%
Minocycline	270	87%	98	61%

**Table 2:** Prevalence of ESBL, AmpC, and CRE among *E. coli* and *K. pneumoniae* in cUTI.

Phenotype	*E. coli*, *n* (%)	*K. pneumoniae*, *n* (%)	Total Prevalence Among *E. coli* (*n* = 406) and *K. pneumoniae* (*n* = 163)
**ESBL**	164 (40.4%)	33 (20.2%)	197 (34.6%)
**AmpC**	10 (2.5%)	6 (3.7%)	16 (2.8%)
**ESBL + AmpC**	14 (3.4%)	1 (0.6%)	15 (2.6%)
**CRE**	7 (1.7%)	28 (17.2%)	35 (6.2%)
**Total**	195	68	263

**Table 3:** **Heat map of antibiotic resistance genes detected within the FOX-resistant *E. coli* using ResFinder.** The figure shows the distribution of antibiotic resistance genes to each antibiotic class. The colored boxes to the right of each strain demonstrate the distribution of antimicrobial resistance genes as follows: aminoglycoside (green), macrolide (light blue), quinolone (peach), folate pathway antagonist (dark gray), tetracycline (dark pink), and beta-lactam (yellow). White boxes indicate absence of these genes.

Isolate	Aminoglycoside				Macrolide	Quinolone			Folate Pathway Antagonist					Tetracycline			Beta-Lactam				
																	AmpC	ESBL	bla_OXA_	bla_TEM_	
	aac(3)-IIa	aadA5	aph(3″)-Ib	aph(6)-Id	mph(A)	aac(6′)-Ib-cr	qnrB4	qnrS1	dfrA7	dfrA14	dfrA17	sul1	sul2	tet(A)	Tet(B)		blaDHA-1	blaCTX-M-15	blaOXA-1	blaTEM-1B	blaTEM-35
0410																					
7659																					
7826																					
8268																					
0495																					
0539																					
0402																					
2536																					
8100																					
9044																					
4976																					

**Table 4:** **Heat map of antibiotic resistance genes detected within the CRE *K. pneumoniae* using ResFinder.** The figure shows the distribution of antibiotic resistance genes to each antibiotic class. The colored boxes to the right of each strain demonstrate the distribution of antimicrobial resistance genes as follows: aminoglycoside (green), quinolone (peach), folate pathway antagonist (dark gray), tetracycline (dark pink), beta-lactam (yellow), and fosfomycin (gold). White boxes indicate the absence of the genes.

Isolate	MLST	Aminoglycoside							Quinolone						Folate Pathway Antagonist						Tetracycline		Beta-Lactam																		Fosfomycin	
		aac(6′)-Ib	aadA1	aadA2	aph(3′)-Ia	aph(3′)-VI	armA	rmtF	aac(6′)-Ib-cr	qnrA	qnrB	qnrS	OqxA	OqxB	dfrA1	dfrA5	dfrA12	dfrA14	sul1	sul2	tet(A)	tet(D)	blaSHV-28	blaSHV-106	blaSHV-40	blaSHV-56	blaSHV-89	blaSHV-11	blaSHV-67	blaTEM-1	blaCTX-M-8	blaCTX-M-15	blaDHA-1	blaOXA-1	blaOXA-9	blaOXA-48	blaOXA-232	blaNDM-5	blaKPC-2	blaOKP-B-3	fosA5	fosA6
4494	ST-2096																																									
1395	ST-2096																																									
4590	ST-2096																																									
9002	ST-2096																																									
4787	ST-2096																																									
5038	ST-2096																																									
0821	ST-2096																																									
2541	ST-2096																																									
2542	ST-2096																																									
9590	ST-2096																																									
8474	ST-2096																																									
8637	ST-2096																																									
1502	ST-231																																									
0403	ST-147																																									
2967	ST-147																																									
0350	ST-147																																									
4279	ST-147																																									
6112	ST-147																																									
7117	ST-147																																									
0845	ST-147																																									
2579	ST-1770																																									
1776	ST-111																																									

## Data Availability

The original contributions presented in this study are included in the article/Supplementary Materials. Further inquiries can be directed to the corresponding author.

## Supplementary Materials

The following supporting information can be downloaded at: https://www.mdpi.com/article/10.3390/diagnostics15091062/s1, Supplementary Document S1: The bio sample accessions numbers; Table S1: Antimicrobial resistance genes and their mechanism of action found in FOX-resistant *E. coli* using CARD; Table S2: Antimicrobial resistance genes and their mechanism of action found in CRE *K. pneumoniae* using CARD.
