# Supplementary material for: Mapping Antimicrobial Resistance in Escherichia coli and Klebsiella pneumoniae from Complicated Urinary Tract Infections in Oman: Phenotypic and Genotypic Insights
Source: Diagnostics (Basel). 2025 Apr 22;15(9):1062. doi: 10.3390/diagnostics15091062 (PMC12071653; doi:10.3390/diagnostics15091062)
Supplement: Supplementary file 1 [file diagnostics-15-01062-s001.zip › Supplementary Document S1.pdf]

Supplementary Document S1

The bio sample accession numbers an links received from the NCBI BioSample Submissions Staff, Bethesda, Maryland USA

Accession	Sample Name	SPUID	Organism	Tax ID	Strain
SAMN47530948	EC1	EC1	Escherichia coli	562	EC1
SAMN47530949	EC2	EC2	Escherichia coli	562	EC2
SAMN47530950	EC3	EC3	Escherichia coli	562	EC3
SAMN47530951	EC4	EC4	Escherichia coli	562	EC4
SAMN47530952	EC5	EC5	Escherichia coli	562	EC5
SAMN47530953	EC6	EC6	Escherichia coli	562	EC6
SAMN47530954	EC7	EC7	Escherichia coli	562	EC7
SAMN47530955	EC8	EC8	Escherichia coli	562	EC8
SAMN47530956	EC9	EC9	Escherichia coli	562	EC9
SAMN47530957	EC10	EC10	Escherichia coli	562	EC10
SAMN47530958	EC11	EC11	Escherichia coli	562	EC11
SAMN47530959	062258_KPAR4494.fasta	062258_KPAR4494.fasta	Klebsiella pneumoniae	573	062258_KPAR4494.fasta
SAMN47530960	062264_KPAR1395.fasta	062264_KPAR1395.fasta	Klebsiella pneumoniae	573	062264_KPAR1395.fasta
SAMN47530961	258881_258881Kb9590.fasta	258881_258881Kb9590.fasta	Klebsiella pneumoniae	573	258881_258881Kb9590.fasta
SAMN47530962	258885_258885Kb0403.fasta	258885_258885Kb0403.fasta	Klebsiella pneumoniae	573	258885_258885Kb0403.fasta
SAMN47530963	258888_258888Kb1502.fasta	258888_258888Kb1502.fasta	Klebsiella pneumoniae	573	258888_258888Kb1502.fasta
SAMN47530964	258890_258890Kb4590.fasta	258890_258890Kb4590.fasta	Klebsiella pneumoniae	573	258890_258890Kb4590.fasta
SAMN47530965	258897_258897Kb9002.fasta	258897_258897Kb9002.fasta	Klebsiella pneumoniae	573	258897_258897Kb9002.fasta
SAMN47530966	258908_258908Kb2967.fasta	258908_258908Kb2967.fasta	Kleb		

siella pneumoniae 573 258908_258908Kb2967.fasta
SAMN47530967 258910_258910Kb0350.fasta 258910_258910Kb0350.fasta Klebsiella pneumoniae 573 258910_258910Kb0350.fasta
SAMN47530968 262660_Kpneumoniae11776.fasta 262660_Kpneumoniae11776.fasta Klebsiella pneumoniae 573 262660_Kpneumoniae11776.fasta
SAMN47530969 262661_Kpneumoniae24279.fasta 262661_Kpneumoniae24279.fasta Klebsiella pneumoniae 573 262661_Kpneumoniae24279.fasta
SAMN47530970 262662_Kpneumoniae38474.fasta 262662_Kpneumoniae38474.fasta Klebsiella pneumoniae 573 262662_Kpneumoniae38474.fasta
SAMN47530971 262663_Kpneumoniae492541.fasta 262663_Kpneumoniae492541.fasta Klebsiella pneumoniae 573 262663_Kpneumoniae492541.fasta
SAMN47530972 262664_Kpneumoniae592542.fasta 262664_Kpneumoniae592542.fasta Klebsiella pneumoniae 573 262664_Kpneumoniae592542.fasta
SAMN47530973 262665_Kpneumoniae65038.fasta 262665_Kpneumoniae65038.fasta Klebsiella pneumoniae 573 262665_Kpneumoniae65038.fasta
SAMN47530974 262666_Kpneumoniae74787.fasta 262666_Kpneumoniae74787.fasta Klebsiella pneumoniae 573 262666_Kpneumoniae74787.fasta
SAMN47530975 262667_Kpneumoniae82579.fasta 262667_Kpneumoniae82579.fasta Klebsiella pneumoniae 573 262667_Kpneumoniae82579.fasta
SAMN47530976 262668_Kpneumoniae96112.fasta 262668_Kpneumoniae96112.fasta Klebsiella pneumoniae 573 262668_Kpneumoniae96112.fasta
SAMN47530977 262669_Kpneumoniae107117.fasta 262669_Kpneumoniae107117.fasta Klebsiella pneumoniae 573 262669_Kpneumoniae107117.fasta
SAMN47530978 262670_Kpneumoniae11845.fasta 262670_Kpneumoniae11845.fasta Klebsiella pneumoniae 573 262670_Kpneumoniae11845.fasta
SAMN47530979 262671_Kpneumoniae128637.fasta 262671_Kpneumoniae128637.fasta Klebsiella pneumoniae 573 262671_Kpneumoniae128637.fasta
SAMN47530980 262672_Kpneumoniae130821.fasta 262672_Kpneumoniae130821.fasta Klebsiella pneumoniae 573 262672_Kpneumoniae130821.fasta

https://www.ncbi.nlm.nih.gov/biosample/47530948

https://www.ncbi.nlm.nih.gov/biosample/47530949
https://www.ncbi.nlm.nih.gov/biosample/47530950
https://www.ncbi.nlm.nih.gov/biosample/47530951
https://www.ncbi.nlm.nih.gov/biosample/47530952
https://www.ncbi.nlm.nih.gov/biosample/47530953
https://www.ncbi.nlm.nih.gov/biosample/47530954
https://www.ncbi.nlm.nih.gov/biosample/47530955
https://www.ncbi.nlm.nih.gov/biosample/47530956
https://www.ncbi.nlm.nih.gov/biosample/47530957
https://www.ncbi.nlm.nih.gov/biosample/47530958
https://www.ncbi.nlm.nih.gov/biosample/47530959
https://www.ncbi.nlm.nih.gov/biosample/47530960
https://www.ncbi.nlm.nih.gov/biosample/47530961
https://www.ncbi.nlm.nih.gov/biosample/47530962
https://www.ncbi.nlm.nih.gov/biosample/47530963
https://www.ncbi.nlm.nih.gov/biosample/47530964
https://www.ncbi.nlm.nih.gov/biosample/47530965
https://www.ncbi.nlm.nih.gov/biosample/47530966
https://www.ncbi.nlm.nih.gov/biosample/47530967
https://www.ncbi.nlm.nih.gov/biosample/47530968
https://www.ncbi.nlm.nih.gov/biosample/47530969
https://www.ncbi.nlm.nih.gov/biosample/47530970
https://www.ncbi.nlm.nih.gov/biosample/47530971
https://www.ncbi.nlm.nih.gov/biosample/47530972
https://www.ncbi.nlm.nih.gov/biosample/47530973
https://www.ncbi.nlm.nih.gov/biosample/47530974
https://www.ncbi.nlm.nih.gov/biosample/47530975
https://www.ncbi.nlm.nih.gov/biosample/47530976
https://www.ncbi.nlm.nih.gov/biosample/47530977
https://www.ncbi.nlm.nih.gov/biosample/47530978
https://www.ncbi.nlm.nih.gov/biosample/47530979
https://www.ncbi.nlm.nih.gov/biosample/47530980
