# Supplementary material for: Mapping Antimicrobial Resistance in Escherichia coli and Klebsiella pneumoniae from Complicated Urinary Tract Infections in Oman: Phenotypic and Genotypic Insights
Source: Diagnostics (Basel). 2025 Apr 22;15(9):1062. doi: 10.3390/diagnostics15091062 (PMC12071653; doi:10.3390/diagnostics15091062)
Supplement: Supplementary file 1 [file diagnostics-15-01062-s001.zip › Supplementary Table S1.pdf]

Supplementary Table S1. Antimicrobial resistance genes and their mechanism of action found in FOX-resistant *E. coli* using CARD.

Mechanism of resistance	Resistant gene	AMR gene family	Drug class	*E. coli* isolate										
				0410	7659	7826	8268	0495	0539	0402	2536	8100	9044	4976
Antibiotic efflux, reduced permeability to antibiotic	marA	Resistance-nodulation-cell division (RND) antibiotic efflux pump, General Bacterial Porin with reduced permeability to beta-lactams	fluoroquinolone antibiotic, monobactam, carbapenem, cephalosporin, glycylcycline, cephamycin, penam, tetracycline antibiotic, rifamycin antibiotic, phenicol antibiotic, penem, disinfecting agents and antiseptics	√	√	√	√	√		√	√	√	√	√
Antibiotic efflux	acrB	RND	fluoroquinolone, cephalosporin, glycylcycline, penam, tetracycline, rifamycin, phenicol antibiotic, disinfecting agents and antiseptics	√	√					√	√	√	√	√
	acrD	RND	aminoglycoside antibiotic	√							√			
	AcrE	RND	fluoroquinolone, cephalosporin, cephamycin, penam		√					√		√	√	√
	AcrF	RND	fluoroquinolone, cephalosporin, cephamycin, penam		√						√			
	AcrS	RND	fluoroquinolone, cephalosporin, glycylcycline, cephamycin, penam, tetracycline, rifamycin, phenicol, disinfecting agents and antiseptics		√							√		

	Escherichia coli acrA	RND	fluoroquinolone, cephalosporin, glycylcycline, penam, tetracycline, rifamycin, phenicol antibiotic, disinfecting agents and antiseptics	√	√						√	√	√	√
	mdtA	RND	aminocoumarin antibiotic		√									
	mdtB	RND	aminocoumarin antibiotic									√		
	mdtN	Major facilitator superfamily (MFS) antibiotic efflux pump	nucleoside antibiotic, disinfecting agents and antiseptics	√	√	√	√				√	√		
	TolC	ATP-binding cassette (ABC) antibiotic efflux pump, MFS, RND	macrolide, fluoroquinolone, aminoglycoside, carbapenem, cephalosporin, glycylcycline, cephamycin, penam, tetracycline, peptide, aminocoumarin, rifamycin, phenicol, penem, disinfecting agents and antiseptics	√	√	√		√		√	√	√	√	√
	cpxA	RND	aminoglycoside, aminocoumarin	√	√				√	√	√	√	√	√
	mdtH	MFS	fluoroquinolone antibiotic		√	√	√	√	√		√	√	√	
	mdtE	RND	macrolide, fluoroquinolone, penam	√							√	√	√	√
	mdtF	RND	macrolide, fluoroquinolone, penam	√							√			
	mdtG	MFS	phosphonic acid antibiotic	√	√					√	√	√	√	√
	mdtO	MFS	nucleoside, disinfecting agents and antiseptics		√									
	kdpE	kdpDE	aminoglycoside antibiotic	√	√						√	√		

	H-NS	MFS, RND	macrolide, fluoroquinolone, cephalosporin, cephamycin, penam, tetracycline	√	√	√	√	√	√	√	√	√	√	√
	emrA	MFS	fluoroquinolone antibiotic	√	√						√			
	emrB	MFS	fluoroquinolone antibiotic	√	√		√	√	√		√	√	√	√
	emrK	MFS	tetracycline antibiotic		√						√	√		
	emrR	MFS	fluoroquinolone antibiotic		√		√	√	√	√		√	√	√
	emrY	MFS	tetracycline antibiotic	√	√	√	√				√			
	mdtM	MFS	fluoroquinolone, lincosamide, nucleoside, phenicol, disinfecting agents and antiseptics			√								
	mdtP	MFS	nucleoside, disinfecting agents and antiseptics	√	√						√	√		
	evgA	MFS, RND	macrolide, fluoroquinolone, penam, tetracycline	√	√		√	√	√	√	√	√	√	√
	evgS	MFS, RND	macrolide, fluoroquinolone, penam, tetracycline	√	√							√		
	msbA	ATP-binding cassette (ABC) antibiotic efflux pump	nitroimidazole antibiotic	√	√					√	√	√	√	√
	gadX	RND	macrolide, fluoroquinolone, penam	√	√						√			
	leuO	MFS	nucleosid, disinfecting agents and antiseptics						√					

	qacEdelta1	MFS	disinfecting agents and antiseptics								√			
	gadW	RND	macrolide, fluoroquinolone, penam						√					
	YojI	ABC	peptide antibiotic	√										√
Antibiotic inactivation	DHA-1	DHA beta-lactamase	cephalosporin, cephamycin	√	√	√	√	√	√	√			√	√
	CTX-M-15	CTX-M beta-lactamase	cephalosporin, penam	√					√		√	√		
	OXA-1	OXA beta-lactamase	carbapenem, cephalosporin, penam								√			
	TEM-1	TEM beta-lactamase	monobactam, cephalosporin, penam, penem			√		√		√		√		
	TEM-190	TEM beta-lactamase	monobactam, cephalosporin, penam, penem									√		
	Mrx	macrolide phosphotransferase (MPH)	macrolide antibiotic	√	√	√	√	√	√	√	√	√	√	√
	mphA	macrolide phosphotransferase (MPH)	macrolide antibiotic	√		√	√	√	√		√	√	√	√
	aadA5	ANT(3")	aminoglycoside antibiotic								√			
antibiotic target replacement	dfrA14	trimethoprim resistant dihydrofolate reductase dfr	diaminopyrimidine antibiotic			√						√		
	sul1	sulfonamide resistant sul	sulfonamide antibiotic	√	√	√	√	√	√	√			√	√
	sul2	sulfonamide resistant sul	sulfonamide antibiotic									√		

	sul3	sulfonamide resistant sul	sulfonamide antibiotic			√								
Antibiotic target alteration	bacA	undecaprenyl pyrophosphate related proteins	peptide antibiotic	√	√	√					√	√		
	PmrF	pmr phosphoethanolamine transferase	peptide antibiotic	√	√					√	√	√	√	√
	ugd	Pmr phosphoethanolamine transferase	peptide antibiotic			√	√							
	eptA	pmr phosphoethanolamine transferase	peptide antibiotic		√									
Antibiotic target protection	QnrB4	quinolone resistance protein (qnr)	fluoroquinolone antibiotic	√	√	√	√	√	√	√			√	√
	QnrS1	quinolone resistance protein (qnr)	fluoroquinolone antibiotic	√		√						√		
