# Supplementary material for: Mapping Antimicrobial Resistance in Escherichia coli and Klebsiella pneumoniae from Complicated Urinary Tract Infections in Oman: Phenotypic and Genotypic Insights
Source: Diagnostics (Basel). 2025 Apr 22;15(9):1062. doi: 10.3390/diagnostics15091062 (PMC12071653; doi:10.3390/diagnostics15091062)
Supplement: Supplementary file 1 [file diagnostics-15-01062-s001.zip › Supplementary Table S2.pdf]

Supplementary Table S2. Antimicrobial resistance genes and their mechanism of action found in CRE *K. pneumoniae* using CARD.

Mechanism of resistance	Resistant gene	AMR gene family	Drug class	*K. pneumoniae* isolate																					
				4494	1395	4590	9002	4787	5038	0821	2541	2542	9590	8474	8637	1502	0403	2967	0350	4279	6112	7117	0845	2579	1776
Reduced permeability to antibiotic	Mdtq	Outer Membrane Porin (Opr)	monobactam, carbapenem, cephalosporin, cephamycin, penam, penem	√	√	√	√	√	√	√	√	√	√	√	√	√	√	√	√	√	√	√	√		√
Antibiotic efflux	LptD	ATP-binding cassette (ABC) antibiotic efflux pump	carbapenem, peptide antibiotic, aminocoumarin antibiotic, rifamycin	√	√	√	√	√	√	√	√	√	√	√	√	√								√	
	oqxA	resistance-nodulation-cell division (RND) antibiotic efflux pump	fluoroquinolone, glycylcycline, tetracycline, diaminopyrimidine antibiotic, nitrofuran	√	√	√	√	√	√	√	√	√	√	√	√	√	√	√	√	√	√	√	√	√	√
	Klebsiella pneumoniae KpnE	small multidrug resistance (SMR) antibiotic efflux pump	macrolide, aminoglycoside, cephalosporin, tetracycline, peptide, rifamycin, disinfecting agents and antiseptics	√	√	√	√	√	√	√	√	√	√	√	√						√	√			
	Klebsiella pneumoniae KpnF	SMR antibiotic efflux pump	macrolide, aminoglycoside, cephalosporin, tetracycline, peptide, rifamycin, disinfecting agents and antiseptics	√	√	√	√	√	√	√	√	√	√	√	√	√	√	√	√	√	√	√	√	√	√

	qacEdelta1	major facilitator superfamily (MFS) antibiotic efflux pump	disinfecting agents and antiseptics	√	√	√	√	√	√		√	√	√		√	√	√	√	√	√	√	√	√		
	tet(D)	MFS antibiotic efflux pump	tetracycline	√	√		√	√	√		√	√	√		√										
Antibiotic inactivation	OXA-232	OXA beta-lactamase	carbapenem, cephalosporin, penam	√	√	√	√	√	√	√	√	√				√						√			
	OXA-48	OXA beta-lactamase	carbapenem, cephalosporin, penam																		√				
	OXA-1	OXA beta-lactamase	carbapenem, cephalosporin, penam	√	√	√	√	√	√	√	√	√	√	√	√							√			
	OXA-9	OXA beta-lactamase	carbapenem, cephalosporin, penam														√						√		
	NDM-5	NDM beta-lactamase	carbapenem, cephalosporin, cephamycin, penam														√	√	√	√			√		
	KPC-2	KPC beta-lactamase	monobactam, carbapenem, cephalosporin, penam																					√	
	OKP-B-3	OKP beta-lactamase	cephalosporin, penam																					√	
	DHA-1	DHA beta-lactamase	cephalosporin, cephamycin																						√
	CTX-M-15	CTX-M beta-lactamase	cephalosporin, penam	√	√	√	√	√	√	√	√	√	√	√	√	√	√	√	√	√	√	√	√		
	CTX-M-8	CTX-M beta-lactamase	cephalosporin, penam																						√

	TEM-1	TEM beta-lactamase	monobactam, cephalosporin, penam, penem	√	√	√	√	√	√		√	√			√	√	√		√	√	√		√		
	SHV-28	SHV beta-lactamase	carbapenem, cephalosporin, penam	√	√	√	√	√	√	√	√	√	√	√	√										
	SHV-11	SHV beta-lactamase	carbapenem, cephalosporin, penam													√	√	√	√	√	√	√	√		√
	SAT-2	streptothricin acetyltransferase (SAT)	nucleoside	√	√	√	√	√	√	√	√	√		√	√										
	mphE	macrolide phosphotransferase (MPH)	macrolide	√	√	√	√	√	√		√	√	√		√		√	√	√	√	√	√	√		
	mphA	MPH	macrolide													√	√	√	√	√	√		√		
	Mrx	MPH	macrolide													√									
	aadA2	ANT(3")	aminoglycoside	√	√	√	√	√	√		√	√	√		√	√									
	AAC(6')-Ib-cr5	AAC(6')-Ib-cr	fluoroquinolone, aminoglycoside	√	√	√			√		√	√			√										
	AAC(6')-Ib	AAC(6')	aminoglycoside													√									
antibiotic target replacement	dfrA12	trimethoprim resistant dihydrofolate reductase dfr	diaminopyrimidine	√	√	√	√	√	√		√	√	√		√										
	dfrA14	trimethoprim resistant	diaminopyrimidine		√	√		√	√		√	√			√										

		dihydrofolate reductase dfr																							
	dfrA5	trimethoprim resistant dihydrofolate reductase dfr	diaminopyrimidine													√	√	√	√	√	√		√		
	sul1	sulfonamide resistant sul	sulfonamide	√	√	√		√	√		√	√	√		√	√	√	√	√	√	√	√	√		√
	sul2	sulfonamide resistant sul	sulfonamide														√	√	√	√	√		√		
Antibiotic target alteration Antibiotic target protection	armA	16S rRNA methyltransferase (G1405)	aminoglycoside	√	√	√	√	√	√		√	√	√		√		√	√	√	√	√	√	√		
	rmtF	16S rRNA methyltransferase (G1405)	aminoglycoside													√									
	QnrA1	quinolone resistance protein (qnr)	fluoroquinolone													√									
	QnrB4	quinolone resistance protein (qnr)	fluoroquinolone																						√

	QnrS1	quinolone resistance protein (qnr)	fluoroquinolone														√	√	√	√	√		√		
	msrE	msr-type ABC-F protein	macrolide, streptogramin	√	√	√	√	√	√		√	√	√		√		√	√	√	√	√	√	√		
